# Neurodegenerative Disorders: Spotlight on Sphingolipids

**DOI:** 10.3390/ijms222111998

**Published:** 2021-11-05

**Authors:** Frida Mandik, Melissa Vos

**Affiliations:** Institute of Neurogenetics, University of Luebeck, Ratzeburger Allee 160, 23562 Lübeck, Germany; Frida.mandik@neuro.uni-luebeck.de

**Keywords:** sphingolipids, ceramide, neurodegeneration, NBIA, Parkinson’s disease

## Abstract

Neurodegenerative diseases are incurable diseases of the nervous system that lead to a progressive loss of brain areas and neuronal subtypes, which is associated with an increase in symptoms that can be linked to the affected brain areas. The key findings that appear in many neurodegenerative diseases are deposits of proteins and the damage of mitochondria, which mainly affect energy production and mitophagy. Several causative gene mutations have been identified in various neurodegenerative diseases; however, a large proportion are considered sporadic. In the last decade, studies linking lipids, and in particular sphingolipids, to neurodegenerative diseases have shown the importance of these sphingolipids in the underlying pathogenesis. Sphingolipids are bioactive lipids consisting of a sphingoid base linked to a fatty acid and a hydrophilic head group. They are involved in various cellular processes, such as cell growth, apoptosis, and autophagy, and are an essential component of the brain. In this review, we will cover key findings that demonstrate the relevance of sphingolipids in neurodegenerative diseases and will focus on neurodegeneration with brain iron accumulation and Parkinson’s disease.

## 1. Introduction

Neurodegenerative disorders (ND) are progressive disorders that impair specific brain areas or neuronal subtypes, accompanied by a gradual increase in signs and symptoms linked to the affected brain areas. A puzzling observation in ND is that in initial, pre- and early clinical stages, only specific neuronal subtypes degenerate, whereas in later, advanced stages, other neuronal subtypes are affected as well, resulting in more comprehensive neuronal degeneration. For example, in Parkinson’s disease (PD), dopaminergic neurons are initially affected, but first symptoms manifest only when the loss is around 70%, followed by broader neurodegeneration [1]. An early diagnosis and slowing down disease progression are key in the treatment of ND; however, there is at least a partial lack of knowledge about the underlying disease pathogenesis, which is a barrier to the development of targeted drugs. While several ND gene mutations have been identified that are causative, the majority of ND remain sporadic, or in the case of PD, both sporadic and genetic forms of the disease exist. Nevertheless, the existence of these genetic forms has enabled the creation of animal models to understand the underlying mechanisms that are affected in ND. Such studies have strongly contributed to our current knowledge and several common molecular mechanisms have been identified to play an important role in the pathogenesis of ND. One interesting observation is the presence of deposits, including proteins, in, e.g., Alzheimer’s disease (AD) and PD, and iron in PD and neurodegeneration with brain iron accumulation (NBIA) [2,3,4,5,6]. It remains, however, unclear if these deposits are the consequence of upstream cellular defects or rather (partially) the cause of the observed signs. Furthermore, mitochondrial dysfunction is a recurring observation in ND, including defects at the level of the energy production and mitophagy, a mitochondria-specific form of autophagy, which has been linked to several NDs [7,8,9,10,11,12]. More recently, lipids (and more specifically sphingolipids) have been shown to play a crucial role in neurodegeneration.

In this review, we will focus on key findings showing the importance of sphingolipids and how alterations in sphingolipids have implications for ND. First, we will give an overview of sphingolipids and their function. Next, we will discuss the importance of sphingolipids in ND using the findings of two specific NDs, namely NBIA and PD.

## 2. Sphingolipids

**Figure 1:**
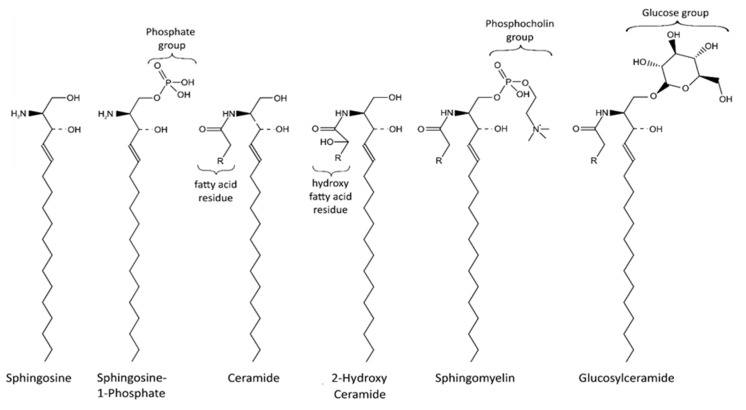
Sphingolipid structure. Sphingolipids contain a sphingoid base (e.g., sphingosine) bound to a fatty acid via an amino bond. Various head groups, including phosphates, phosphocholine and saccharides, can bind to the sphingoid base. The hydrophobic tail and hydrophilic head make sphingolipids amphipathic.

**Figure 2:**
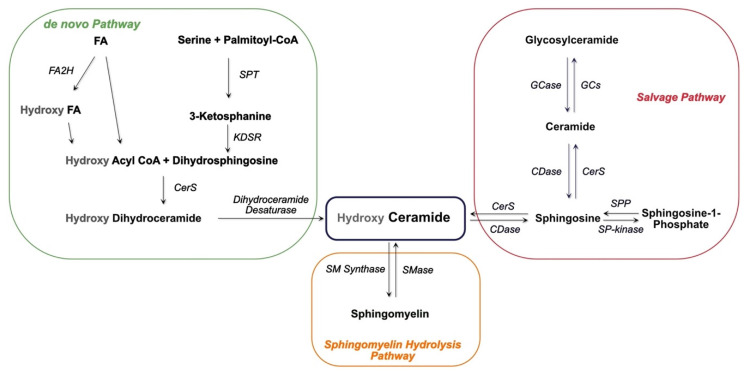
Scheme of ceramide synthesis. Ceramide is synthesized via three distinct pathways, the de novo, the salvage and the sphingomyelin hydrolysis pathways. FA: fatty acid; FA2H: fatty acid 2 hydroxylase; SPT: serine palmitoyl transferase; KDSR: 3-ketosphingosine reductase; CerS: ceramide synthase; GCase: glucocerebrosidase; GCs: glucosyl ceramide synthase; CDase: ceramidase; SP-kinase: sphingosine kinase; SPP: sphingosine-1 phosphatase.

Sphingolipids are an important class of lipids that comprise a fatty acid that is attached to a sphingoid long-chain base (Figure 1). The sphingoid bases are aliphatic amino alcohols [13]. When a fatty acid binds to the amino group, ceramides are formed, which are the base of all sphingolipids. The incorporated fatty acids are very diverse. In addition to different carbon chain lengths, variations in saturation and hydroxylation exist [14]. Furthermore, sphingolipid groups attach different head groups to the first carbon atom of the sphingoid base. This makes the lipids amphipathic, meaning that they have both a hydrophobic and a hydrophilic region. A ceramide that consists of only one hydroxyl head group has a limited hydrophobicity. The amphipathic effect is stronger in more complex sphingolipids such as sphingomyelin and glycosphingolipids that bind phosphatidylcholine and saccharides, respectively (Figure 1) [14]. Ceramide metabolism occurs via three distinct pathways (Figure 2). The first pathway, de novo synthesis, occurs in the endoplasmic reticulum (ER). Here, dihydrosphingosine is formed from serine and a palmitoyl-CoA. Once dihydrosphingosine binds to a fatty acyl CoA, dihydroceramide is formed. This step is catalyzed by ceramide synthases. In humans, six different ceramide synthases are described that use different fatty acids as substrates. The dihydroceramide is subsequently converted into ceramide [15,16]. The second pathway is the salvage pathway, which takes place in the lysosomes. In this pathway, ceramides are obtained by the catabolism of complex sphingolipids. The sphingoid base is formed, which is reacetylated to ceramides [16,17]. The third and most rapid metabolic pathway is sphingomyelin hydrolysis, which occurs in the Golgi apparatus. Sphingomyelin is degraded to ceramide and phosphatidylcholine [16,18]. Ceramide metabolism is tightly regulated by enzyme expression, post-translational modifications, and allosteric mechanisms [14]. Sphingolipids are bioactive lipids involved in numerous cellular processes. The various sphingolipids fulfill different roles and may have antagonistic effects. Ceramide regulates cell stress response and inhibits cell growth, as well as promoting apoptosis, cell senescence, and autophagy, in particular mitophagy [19,20]. Several ceramides have been associated with mitochondrial dysfunction, including reduced mitochondrial respiratory chain, increased reactive oxygen species (ROS), and reduced mitochondrial membrane potential [16]. The ceramide precursor sphingosine has similar functions. In addition to the regulation of actin cytoskeleton and endocytosis, it induces cell cycle arrest and stimulates apoptosis [19,21]. Sphingosine-1-phosphate (S1P), a degradation product of ceramide, exhibits antagonistic effects on ceramide and sphingosine. It promotes cell growth and survival, and prevents apoptosis [14,19,21,22]. Furthermore, it regulates cell migration [19]. The more complex sphingomyelin is part of the myelin sheath that surrounds axons and it stimulates axonal growth and neuronal transmission. Additionally, it is required for presynaptic plasticity and neurotransmitter receptor localization [23,24,25]. Another large and complex group of sphingolipids are glycosphingolipids. They are involved in post-Golgi transport and cell differentiation. Studies have revealed hydroxylated galactosyl ceramides to be essential for myelin structure, function, and maintenance. However, they are not essential for myelin formation [26]. Hydroxy sphingolipids, in general, are lipids of which the fatty acid residue is hydroxylated [27]. Their function has also been linked to the epidermal permeability barrier, axonal development, the cAMP-dependent signaling pathway that regulates the cell cycle, and apoptosis [26,27].

## 3. Neurodegeneration with Brain Iron Accumulation

NBIA is a rare, heterogeneous group of genetic NDs characterized by abnormal brain iron accumulation. Other common findings are cerebellum and cortex atrophy [5,6]. The most frequent clinical findings are extrapyramidal symptoms such as dystonia, parkinsonism, and choreoathetosis. Furthermore, cognitive impairment and psychiatric disturbance are often observed [28].

To date, ten genes have been identified to be classic NBIA genes [6]. The most common form of NBIA that occurs in 35–50% of NBIA cases is pantothenate kinase-associated neurodegeneration (PKAN), which arises from mutations in *pantothenate kinase 2* (*PANK2*). The second largest group, constituting 20% of NBIA patients, is PLA2G6-associated neurodegeneration (PLAN) which is caused by mutations in *phospholipase A2* (*PLA2G6*). Mitochondrial membrane protein-associated neurodegeneration (MPAN), induced by mutations in *chromosome 19 open reading frame 12* (*C19orf12*), was identified in 6–10% of NBIA patients, and ß-propeller-associated neurodegeneration (BPAN), caused by a mutation in *tryptophan-aspartic-acid repeat domain 45* (*WDR45*), was identified in 1–2% of NBIA cases [28]. Fatty acid hydroxylase-associated neurodegeneration (FAHN) (with *fatty acid 2 hydroxylase* (*FA2H*) mutation), neuroferritinopathy (with *Ferritin* (*FTL1*) mutation), aceruloplasminemia (with *ceruloplasmin* (*CP*) mutation), Woodhouse–Sakati syndrome (with *DB1* and *Cul4-associated factor 17* mutation), Kufor–Rakeb syndrome (with *ATPase cation transporting 13A2* mutation), and COASY protein-associated neurodegeneration (with *CoA synthase* mutation) are rare diseases affecting less than 1% of NBIA cases (Table 1) [28]. In addition, many patients present with typical NBIA symptoms; however, to date, no disease-causing mutations have been identified.

**Figure 3:**
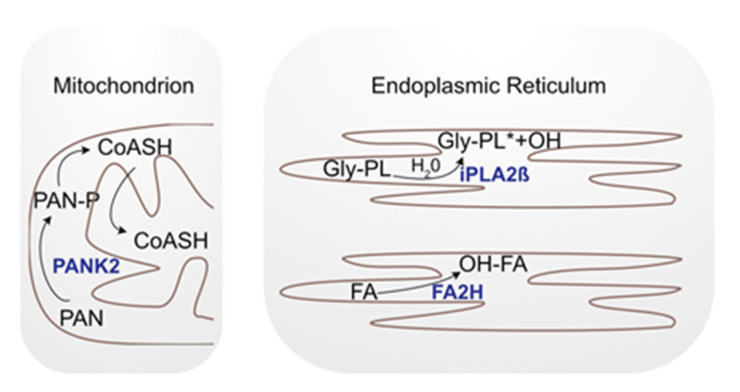
Simplified scheme of the cellular localization of NBIA genes. PANK2 is localized in the mitochondria where it phosphorylates pantothenate (PAN), a precursor of a Coenzyme A group (CoASH). FA2H and iPLA2-VIA are localized in the ER. Fatty acid hydroxylase (FA2H) adds a hydroxyl group (OH) to fatty acids (FA) and iPLA2ß hydrolyzes phospholipids (Gly-PL: glycerophospholipids; *: hydrolyzed).

Although the key finding of NBIAs is iron deposition in the brain, it has not yet been established whether this is a causal or a symptomatic feature of the disease. Iron reduction in PKAN patients showed no improvement in clinical findings [29]. Furthermore, only two NBIA-associated proteins (ceruloplasmin and ferritin light chain) can be directly linked to iron metabolism. The other proteins are localized either to mitochondria (4 out of 10), the ER (2 out of 10), the nucleus (1 out of 10), or lysosomes (1 out of 10), and are associated with apoptosis, autophagy, CoA synthesis, lipids, myelin metabolism, and membrane modulation [30]. Nonetheless, the exact function of each protein has not yet been elucidated. However, the localization and associated functions suggest a link to altered sphingolipid metabolism. We will discuss these interactions in more detail in the following section using FAHN, PKAN and PLAN as examples (Figure 3).

### 3.1. Fatty Acid Hydroxylase-Associated Neurodegeneration and Sphingolipids

FAHN is a small group of NBIA disorders that are inherited in an autosomal recessive manner. The first symptoms appear in early childhood and the most common clinical findings are spasticity, cognitive impairment, ataxia, and dystonia [30]. It is caused by a loss-of-function mutation in *FA2H* that encodes the enzyme fatty acid 2-hydroxylase, which is localized in the ER. The protein is involved in the synthesis of sphingolipids containing 2-hydroxylated fatty acids [31] providing a direct link between affected sphingolipid metabolism and the disease. Nonetheless, the etiology of the disease remains enigmatic. White matter, which is altered in FAHN patients, consists mainly of myelin [32,33]. The sphingolipids galactosyl ceramides and sulfatides represent about 30% of the lipids of myelin and consist largely of 2-hydroxy fatty acids [34]. A 3–20-fold increase in 2-hydroxy ceramides and 2-hydroxy fatty acids upon the expression of human FA2H in COS-7 cells were observed [31]. However, how these alterations result in FAHN-related phenotypes remains unresolved. In an attempt to unravel the pathology of FAHN, model organisms were developed that mimic the disease. Studies of FA2H-deficient mice presented with typical FAHN symptoms, including demyelination, impaired cerebellum function, and the degradation and swelling of axons [35,36]. In *C. elegans*, a FAHN model showed inhibition of lipid droplet formation [37]. Unfortunately, no direct disease mechanism was identified; however, in recent years, there has been an increasing amount of publications showing that FA2H plays an important role in cancer by its stimulatory effect on cell growth [38,39,40,41,42]. Moreover, FA2H expression has been shown to induce apoptosis in gastric cancer cells [42], indicating that alterations to these hydroxy sphingolipids affect cell growth and apoptosis.

### 3.2. Pantothenate Kinase-Associated Neurodegeneration and Sphingolipids

PKAN is inherited in an autosomal recessive fashion and can be subdivided into *“*classic*”*, *“*atypical*”* and *“*intermediate*”* types, depending on the age of onset and disease progression [43]. Typical symptoms are dystonia and cognitive dysfunction [43]. *PANK2* encodes the enzyme pantothenate kinase 2, which is one of four pantothenate kinase proteins. It is localized to mitochondria where it phosphorylates the coenzyme A precursors pantothenate, *N*-pantothenoyl-cysteine, and pantetheine [44,45]. An examination of PKAN patient-derived plasma showed elevated lactate levels indicative of mitochondrial dysfunction [46]. This was confirmed by experiments using knock-out (KO) mice, knock-down (KD) Drosophila and iPSC-derived cortical neurons that showed altered mitochondrial membrane potential, swollen mitochondria and increased ROS [47,48,49]. In addition, Coenzyme A, which is directly involved in lipid metabolism, was reduced upon the KD of PANK2 in a fruit fly, which is consistent with the increased pantothenate levels found in the plasma of patients [46,47]. Moreover, experiments were performed to investigate the lipid composition of plasma and red blood cells of PKAN patients. Glycerolipids, the sphingolipid C16:1 and sphingomyelin were reduced. Interestingly, the analysis of the distribution of the different lipids in the membrane of red blood cells showed that the molar percentage of sphingomyelin was increased compared to that of the other lipids [46,50]. Thus, studies suggest an interaction between the mitochondrial PANK2 and altered sphingolipids metabolism. However, the nature of this link and how this results in ND are yet to be clarified.

### 3.3. Phospholipase A2 Group VI and Sphingolipids

PLAN is inherited in an autosomal recessive manner. It is an umbrella term for various disorders as a consequence of a plethora of mutations in the *PLA2G6* gene [51]. The different PLAN types depend on the age of onset and severity of symptoms and can be divided into classic infantile neuroaxonal dystrophy (INAD), atypical neuroaxonal dystrophy (NAD) and PLA2G6-related dystonia-parkinsonism [43]. However, no direct correlation between the mutations and PLAN types has been identified [52]. Neurological examinations of INAD and atypical NAD patients often reveal cerebellar atrophy, iron deposition in the globus pallidus, abnormal axons (spheroid bodies), and Lewy bodies, as well as hyperphosphorylated tau [53,54,55].

*PLA2G6* encodes calcium-independent phospholipase A2 ß (iPLA2β), an enzyme that catalyzes the hydrolysis of glycerophospholipids [51]. It is involved in cell proliferation, inflammation, immune responses, apoptosis, and the modulation of phospholipids [52,56]. iPLA2β is widely distributed in the human body and is particularly abundant in the brain, dendrites and axon terminals [57]. Different models have been established to further investigate the function of iPLA2ß. Studies with various iPLA2ß KO mice and fly models revealed motor defects, spheroid formation, autophagic dysfunction, the degradation of axons and synapses, mitochondrial abnormalities, including defects at the inner mitochondrial membrane potential, and oxidative stress [58,59,60,61,62]. Similar results were observed in fibroblasts derived from patients suffering from INAD and PLA2G6-related dystonia-parkinsonism [62]. In addition, a KD model of iPLA2β in zebrafish was created which, in addition to axonal degeneration, showed increased alpha-synuclein expression and the degradation of dopaminergic and motor neurons in iPLA2-ß downregulated neurons [63].

iPLA2ß catalyzes the hydrolysis of glycerophospholipids, suggesting that alterations in phospholipid metabolism make up the underlying molecular mechanism of PLAN. Studies in a KO mouse model indeed showed an altered phospholipid composition in the spinal cord. [59]. However, this could not be identified in a mutant Drosophila model [64]. Studies on lipid alterations mediated by a mutation in iPLA2ß revealed increased lipid peroxidation levels in a KO Drosophila model. Lowering the lipid peroxidation levels rescued the accompanying motor symptoms in a fly model and the mitochondrial membrane potential in patient-derived fibroblasts [62]. Remarkably, the loss of iPLA2ß in flies leads to an accumulation of ceramide. Furthermore, iPLA2-VIA binds to Vps26 and Vps35, two subunits of the retromer complex, which is important for lipid recycling. Hence, the loss of iPLA2-VIA resulted in impaired retromer function, preventing sphingolipids from being transported to the plasma membrane. Instead, sphingolipids are converted to ceramide in lysosomes. This results in ceramide accumulation, which has a toxic effect on the cell [64].

## 4. Parkinson’s Disease

PD is the second most common ND after AD that results in motor symptoms, bradykinesia and rigidity as a consequence of the loss of dopaminergic neurons. PD is characterized by the presence of Lewy bodies, the primary protein structure of which is composed of alpha-synuclein protein aggregates [3].

Like many NDs, PD is a mostly sporadic disease; however, genes have been identified to be causative when mutated, including *SNCA*, *VPS35*, and *LRRK2*, resulting in autosomal dominant forms of PD, and *Parkin* and *PINK1* to be the most common genes mutated in autosomal recessive forms of PD [3,65]. Despite the identification of these genes, there is no complete understanding of the etiology of PD, which explains the limited efficacy of therapeutics. However, similar mechanisms are present in both sporadic and genetic forms of the disease. Studies on drug abusers with parkinsonism have hinted towards defects at the level of the mitochondrial electron transport chain, which was later confirmed in patients and animal models [66,67,68,69,70]. Furthermore, PINK1-dependent phosphorylation is required for an efficient ETC, and the stimulation of the ETC can alleviate signs in pink1- and drug-related PD animal models [71,72,73]. Additionally, PINK1 exerts a (parallel) function together with Parkin that was first identified in Drosophila [74,75]. These findings were later confirmed in a cellular model in which PINK1–Parkin-mediated mitophagy was identified [76,77,78]. Interestingly, mitochondrial symptoms have been observed in other genetic PD models, including alpha-synuclein-dependent models [79], suggesting that mitochondrial dysfunction plays an important role in the pathogenesis of PD; however, the underlying mechanisms of how mitochondrial dysfunction results in neurodegeneration remains enigmatic. A more recently identified pathway that is linked to PD is the endo-lysosomal pathway, in which the autosomal dominant PD-related genes play a major role [70]. Alpha-synuclein and LRRK2 are linked to Rab proteins, which are important in the endo-lysosomal pathway [80,81]. In addition, LRRK2 is a kinase that, amongst other functions, phosphorylates recycling vesicles [82,83,84,85,86]. Furthermore, VPS35 is a subunit of the retromer complex that is involved in the recycling of proteins. Remarkably, alpha-synuclein and LRRK2 have been linked to sporadic forms of PD, highlighting the importance of this pathway. While our knowledge of the affected pathways and mechanisms in PD has strongly improved, the full mechanistic pathway resulting in PD and its accompanying signs and symptoms remains elusive. However, an increasing number of studies show the importance of (sphingo-)lipids in the etiology of PD.

Parkinson’s Disease and Sphingolipids

**Figure 4:**
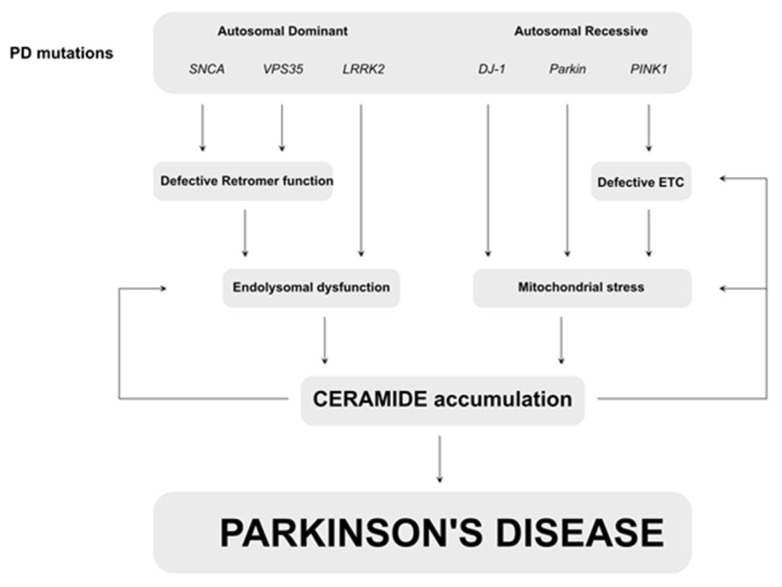
Simplified scheme of mechanisms underlying PD that converge to ceramide accumulation. Mutations in *SNCA* and *VPS35* result in defective retromer function followed by endolysosomal dysfunction. Loss of LRRK2 leads to similar defects. Defects at the level of the endo-lysosome result in accumulation of ceramide. The autosomal recessive PD-related genes *Parkin*, *DJ-1* and *PINK1* cause mitochondrial defects via the increased mitochondrial stress which result in ceramide accumulation. Ceramide accumulation has feedback loops that worsen endolysosomal function and mitochondrial stress, creating a viscious cycle that finally results in Parkinson’s disease.

Recent studies found lipids to be present together with mitochondria and alpha-synuclein aggregates in Lewy bodies [87], which is indicative of an important correlation between lipids and mitochondria in relation to PD. Indeed, several lipids have been identified to play a role in PD pathogenesis, including sterol regulatory element-binding transcription factor 1 (SREBF1), cardiolipin, and phosphatidylserine [88,89,90]. In addition, alterations in the sphingolipid pathway play a protective role against alpha-synuclein aggregates in a *C. elegans* model of PD [91]. Remarkably, recent studies have identified the sphingolipid ceramide to be a key factor in PD pathogenesis. Elevated levels of ceramide were found in the plasma of PD patients and several animal models, while another study found lower ceramide levels and a shift to shorter ceramide species in postmortem brains of sporadic PD patients [64,92,93,94,95]. While contradictory at first sight, ceramide function is defined by its cellular localization and species length [96], providing a possible explanation for this discrepancy. Furthermore, mutations in *GBA* (Figure 2) are the most common genetic risk factor for PD [97,98]. *GBA* encodes Glucocerebrosidase (GCase), which forms ceramide through the hydrolysis of glucosylceramide [97,99]. In homozygous conditions, mutations in *GBA* cause Gaucher’s disease, a lysosomal storage disease [100]. Heterozygously, mutant GCase interacts with alpha-synuclein, resulting in a worsening of the observed signs in PD animal models [97]. In addition, the overexpression of human mutant *GBA* in flies results in locomotion defects and neurodegeneration [101,102,103], further linking ceramides and GCase to PD. Studies using animal PD models have further elaborated on the contribution of sphingolipids to PD. The autosomal dominant PD genes *VPS35* and *alpha-synuclein* are linked in a complex, together with iPLA2ß (see above), to stimulate retromer function, the promoting recycling of proteins and lipids [64]. Defective retromer function, due to the loss of VPS35 or overexpression of alpha-synuclein, inhibits the recycling of lipids, resulting in ceramide accumulation followed by the expansion of lysosomes and neurodegeneration. Lowering of ceramide levels or stimulation of the retromer function can alleviate these phenotypes [64]. LRRK2 plays a role in the recycling of vesicles, suggesting that LRRK2 similarly affects this pathway. Furthermore, LRRK2 KO mice result in altered sphingolipids, including increased ceramide levels in the brain [94], further supporting the important function of sphingolipids in the endo-lysosomal pathway, resulting in PD. More recently, we linked the autosomal recessive PD genes PINK1 and Parkin to ceramide, where ceramide levels are increased in isolated mitochondria [104] to induce ceramide-mediated mitophagy [105]. The increased ceramide levels negatively correlate with ETC efficiency and thus initiate a vicious cycle meaning that an increase in ceramide levels further elevates the requirement for mitochondrial clearance [104]. Thus, studies in animal models centralize ceramide as a common denominator on which autosomal dominant and recessive forms of PD converge (Figure 4).

## 5. Conclusions

It has been decades since lipids were first implicated in NDs [106,107,108], but only over the last few years has their importance for ND pathogenesis garnered increasing attention. Recent findings suggest that sphingolipids play an important role in the underlying mechanisms of NDs, as we discussed for NBIA and PD. Thus, sphingolipids appear to be an interesting therapeutic target in ND. Nutrition provides many lipids, some of which cannot be synthesized by the body. Thus, in addition to drugs, a diet controlling the specific affected sphingolipids might provide a beneficial effect on patients too. For instance, dairy products are linked to an increased risk or progression of PD [109]. Interestingly, the sphingolipids in milk include ceramides [110] that aggravate the PD-related symptoms [104]. Furthermore, a high-fat diet results in increased lipid peroxidation and oxidative stress. However, studies that focus on manipulating the intake of sphingolipids via nutrition and their effect on ND-related pathways need to be further explored.

In this review, we limited our discussion of the importance of sphingolipids to NBIA and PD; however, numerous common defects have been identified in the pathogenesis of ND. Among these, mitochondrial dysfunction and deposits in the endo-lysosomal pathway are the most commonly observed, suggesting that similar pathways are important in the development of different NDs. Thus, sphingolipids can serve as a link between mitochondrial dysfunction and a defective endo-lysosomal pathway, resulting in neurodegeneration.

## Figures and Tables

**Table 1:** Molecular features of PD and NBIA genes. PKAN: pantothenate kinase-associated neurodegeneration; PLAN: PLA2G6-associated neurodegeneration; MPAN: mitochondrial membrane protein-associated neurodegeneration; BPAN: ß-propeller-associated neurodegeneration; FAHN: fatty acid hydroxylase-associated neurodegeneration; CoPAN: COASY protein-associated neurodegeneration; PD: Parkinson’s disease; *SNCA: Synuclein Alpha*; *VPS35*: *vacuolar protein sorting 35*; *LRRK2*: *leucine-rich repeat kinase 2*; *PINK1*: *PTEN-induced kinase 1*; ER: endoplasmic reticulum; MAM: mitochondria associated-membrane.

Disease	Gene	Protein Localization	Pathway
**PKAN**	*PANK2*	Mitochondrium	Coenzyme A Biosynthesis
**PLAN**	*PLA2G6*	Mitochondrium, ER and Cytosol	Lipid Metabolism
**MPAN**	*C19orf12*	Mitochondrium, ER and MAM	Lipid Metabolism
**BPAN**	*WDR45*	ER	Autophagy
**FAHN**	*FA2H*	ER	Lipid Metabolism
**Neuroferritinopathy**	*FTL1*	Cytoplasm	Iron Homeostasis
**Aceruloplasminemia**	*CP*	Plasma Membrane	Iron Homeostasis
**WoodHouse–Sakati Syndrome**	*DCAF17*	Nucleolus	Unknown function
**Kufor–Rakeb Syndrome**	*ATP13A2*	Mitochondrium and Lysosome	Autophagy
**CoPAN**	*COASY*	Mitochondrium and Cytosol	Coenzyme A Biosynthesis
**PD**	*SNCA*	ER and Golgi Apparatus	Endo-Lysosomal Pathway
	*VPS35*	Retromer Complex	Endo-Lysosomal Pathway
	*LRRK2*	ER and Golgi Apparatus	Endo-Lysosomal Pathway
	*DJ-1*	Mitochondrium	Mitochondrial Function
	*Parkin*	Mitochondrium and Cytosol	Mitochondrial Function and Mitophagy
	*PINK1*	Mitochondrium	Mitochondrial Function and Mitophagy

## Data Availability

No new data were created or analyzed in this study. Data sharing does not apply to this article.
